# Supplementary material for: Causal Relationship between Aging and Anorexia Nervosa: A White-Matter-Microstructure-Mediated Mendelian Randomization Analysis
Source: Biomedicines. 2024 Aug 16;12(8):1874. doi: 10.3390/biomedicines12081874 (PMC11351342; doi:10.3390/biomedicines12081874)
Supplement: Supplementary file 1 [file biomedicines-12-01874-s001.zip › Supplementary Figure/Figure S7/Single SNP plot of MR analysis for ubm-b-1815 to AN.pdf]

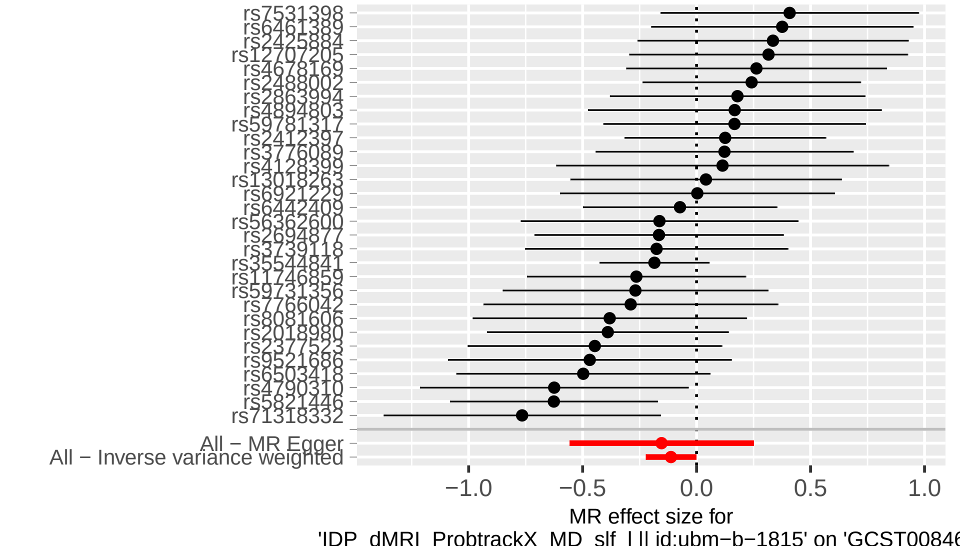
rs7531398
rs6461389
rs2425884
rs12707205
rs4678169
rs2488002
rs2863994
rs4894803
rs59781317
rs2412397
rs3776089
rs4128399
rs13018263
rs6921229
rs6442409
rs56362600
rs2694877
rs3739118
rs35544841
rs11746859
rs59731356
rs7766042
rs8081606
rs2018980
rs2377523
rs9521686
rs6503418
rs4790310
rs5821446
rs71318332
All − MR Egger
All − Inverse variance weighted
−1.0
−0.5
0.0
0.5
1.0
MR effect size for
'IDP_dMRI_ProbtrackX_MD_slf_l || id:ubm−b−1815' on 'GCST0084
